# When insulin degludec enhances quality of life in patients with type 2 diabetes: a qualitative investigation

**DOI:** 10.1186/s12955-018-0883-1

**Published:** 2018-05-03

**Authors:** James Weatherall, William H. Polonsky, Sally Lanar, Naomi Knoble, Jonas Håkan-Bloch, Elisabeth Constam, Athena Philis-Tsimikas, Alexia Marrel

**Affiliations:** 1 Novo Nordisk Inc, 800 Scudders Mill Road, Plainsboro, NJ USA; 2 Behavioral Diabetes Institute, San Diego, CA USA; 3 University of California, San Diego, CA USA; 4 Mapi Group, Lyon, France; 5 Mapi Group, Boston, MA USA; 6 Novo Nordisk A/S, Soeborg, Denmark; 7 Winterthur, Switzerland; 8 Scripps Whittier Diabetes Institute, La Jolla, CA USA

**Keywords:** Insulin degludec, Type 2 diabetes, Health-related quality of life, Patient interviews, Patient focus groups, qualitative research

## Abstract

**Background:**

Anecdotal reports suggest that insulin degludec (IDeg) may offer unique health-related quality of life (HRQoL) benefits. As the nature of these benefits remain unclear, this study utilized qualitative research methods to investigate and elucidate the experience of “feeling better” after initiating IDeg.

**Methods:**

Twenty adults with type 2 diabetes (T2D) who reported “feeling better” on IDeg for > 3 months participated in 90-min interviews. One focus group and nine telephone interviews were conducted at two sites in the United States (US) and one focus group was conducted in Switzerland. Patients were ≥ 18 years of age, did not take mealtime insulin, and had switched to IDeg from another basal insulin. Discussions were audio-recorded, transcribed and translated (Swiss German). Utilizing grounded theory, transcripts were analyzed by sorting quotes into concepts using thematic analysis.

**Results:**

Participants' mean age was 66 years and the average duration of T2D was 17.6 years. Mean duration of IDeg use was 1.45 years. Four major factors were identified as key contributors to patients’ sense of “feeling better”: 1) reduced sense of diabetes as burdensome and requiring excessive attention; 2) enhanced feelings of adaptability and freedom; 3) heightened sense of security, especially regarding concerns about hypoglycemia; and 4) greater sense of physical well-being (greater energy/less fatigue). Content saturation was achieved. Generally, patients from the US sites were more focused on medical results than Swiss patients, who were more likely to identify IDeg’s effect on overall HRQoL. A limitation of the study was that the population was primarily white, > 60 and otherwise healthy (no comorbid physical or mental condition).

**Conclusions:**

A group of patients with T2D, who had switched to IDeg from another basal insulin, reported HRQoL benefits which were attributed to both diabetes-specific improvements (feeling less burdened by day-to-day diabetes demands) and non-specific gains (greater energy). The conclusions may have limited transferability due to the characteristics of the sample population and further research is needed.

## Background

Initiation of basal insulin in adults with type 2 diabetes (T2D) has been linked to improvements in health-related quality of life (HRQoL) [1–5], though anxiety regarding self-injection and complicated dosing regimens continues to burden patients [6]. Published data demonstrates improvement in glycemic control and well-being (vitality and general mood) in patients receiving long-acting basal insulin therapy [5]. However, hypoglycemia related to T2D treatment has a significant negative impact on HRQoL and productivity in patients with diabetes [7, 8]. A study by Evans, et al. [1] suggested that the ability to dose basal insulin in an adaptable manner (by changing the time of administration from day-to-day according to patient requirements) enhances HRQoL, which could potentially make adherence to treatment easier. Limited published information is available on how the initiation of insulin impacts HRQoL and how different insulins affect HRQoL.

Anecdotal evidence suggests that there may be unique and potentially important HRQoL benefits associated with the initiation of insulin degludec (IDeg), often reported by many patients as “feeling much better,” though the actual meaning of such claims remain unspecified and unverified. If true, these presumed benefits may be related—at least “partially—to” increases in perceived vitality and/or reductions in bodily pain [3, 5], but the actual nature of these benefits remains unclear and further study and clarification of these potential associations are needed. This study utilized qualitative research methods to investigate and elucidate the experience of “feeling better” after initiating IDeg.

## Methods

**Fig. 1:**
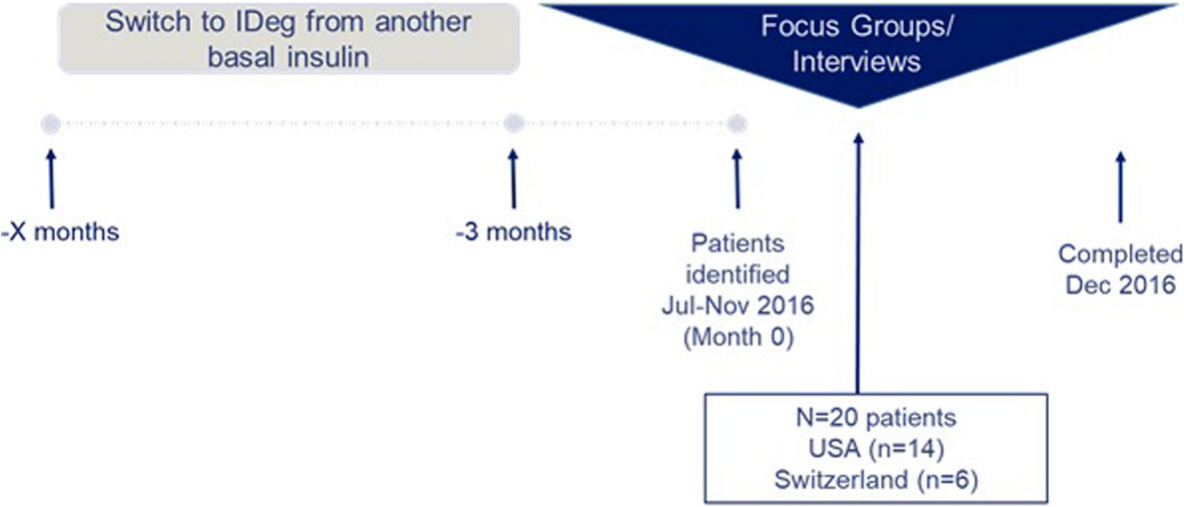
Cross-sectional study design. Legend: IDeg, insulin degludec

This qualitative research study was conducted at three centers (two in the US and one in Switzerland). Sites were chosen in the US and Switzerland because they fulfilled the criteria that were needed to conduct the qualitative study. These criteria included having IDeg on the market, willingness of sites to participate, patient recruitment capability and sites with anecdotal evidence of patients “feeling better.” Central Institutional Review Board approval was obtained for US sites from New England IRB (20160797) and an Ethics waiver from The Zurich Canton Ethics Committee (2016–00861) for the site in Switzerland. Written informed consent was given by all patients. The study design for the qualitative assessment of patients “feeling better” on IDeg was set up as follows: Sites in the US and Switzerland that had patients on IDeg were contacted. Among the three sites that agreed to participate, investigators referred a consecutive sample of patients on IDeg who were requested to fill out a screening questionnaire. Inclusion and exclusion criteria were the same for both countries. Adult patients (≥18 years of age) with T2D taking IDeg for at least 3 months and who reported “feeling better” on IDeg and were not taking a meal-time insulin were included in the study and invited to participate in focus groups or one-on-one interviews. Patients excluded were those with a physical or mental comorbid condition or history of drug or alcohol problems that might have limited their ability to participate in the study. Also excluded were patients who were currently enrolled in a clinical trial or an interventional study and those who, in the opinion of the clinician, would not comply with the study requirements (see Fig. 1 for a graphic illustration of the study design).

The focus group/interviews focused on: 1) How patients using IDeg feel better than they did prior to using IDeg; 2) Explain what “feeling better” means and what has changed? For example, has IDeg contributed to an improvement in their mood, activities of daily living, sleep, experience of hypoglycemia, general well-being, etc.; and 3) What, specifically, about using IDeg do patients think has helped them to feel better? Specific relevant details on the interview questions are provided in Table 1.

**Table 1:** Focus group discussion and telephone interview questions

Interview Questions
1. How would you describe any effect of IDeg on your daily routine?
• Flexible dosing? • Changes in frequency of blood sugar checking? • Is your daily life the same, easier, more complex? • Changes in level of concern/anxiety about hypoglycemia? • Changes in confidence for managing medications?
2. How would you describe the overall effect of IDeg on your life?
• Differences in before/after taking IDeg? • Differences between the effect of IDeg and other medications you took before? • Managing your diabetes? • Changes in frequency of hypoglycemia?
3. If you feel better when taking IDeg, can you tell us more about what “feeling better” means to you? How can you tell that you feel better?
• Changes in social life (interactions with family/friends) • Changes in work life (interactions with co-workers/ability to do work) • Feeling less anxious/worried • Changes in energy/mood (day/night; concentration; less tired) • Changes in sleep (more/less; changes in blood sugars at night) • Changes in ability to exercise (more/less; confidence) • Feeling “friskier” (change in sex drive)? • Paying less for medication

IDeg, insulin degludec

Case report forms were completed by the investigator (selection criteria, diagnosis, last basal insulin, diabetes-related treatment [oral and injectable], comorbidities). A socio-demographic form was completed by patients and included the following: gender, age, highest level of education, work status, time since T2D diagnosis, time since first basal insulin treatment, and time since initiation of IDeg treatment. Race and ethnicity were only collected for US patients but not for Swiss patients due to cultural reasons. There were six Swiss patients and five US patients at the focus groups. For those unable to participate in focus groups (*n* = 9, all in the US), individual phone interviews (n = 9) were conducted. Focus groups (approximately 90 min) and phone interviews (approximately 30 min) were conducted by skilled moderators trained in patient-reported outcome and semi-structured interview techniques. The interviews and focus groups were audio-recorded with participants’ permission, transcribed and analyzed. The Swiss German transcript was translated into English. Socio-demographic and clinical data were summarized using descriptive statistics. Discussion groups and interviews were analyzed qualitatively according to the principles of saturation [9–11]: data collection continues until further data collection produces minimal or no new information to further confirm or challenge. Concept coding was carried out using Atlas.ti software, version 7.0. Qualitative analyses were conducted on all transcripts by sorting quotes into concepts using thematic analysis methods based on grounded theory principles [12, 13]. A team of three analysts were involved in the coding process.

## Results

A sample of 20 basal insulin-treated T2D patients met the inclusion criteria and were recruited from the three sites; two sites in US (*n* = 14) and one in Switzerland (*n* = 6) (Table 2). The mean age of patients was 66 years and the average duration of T2D was 17.6 years. The average time since first basal insulin was 5.9 years and mean IDeg use was 1.45 years. The majority (95%) of patients had at least high school education and more than half (55%) had some college or a bachelor’s degree. Approximately half (55%) of patients were not working, the majority of whom were retired.

**Table 2:** Patient demographics

Characteristic		Overall *N* = 20	US *N* = 14	Switzerland *N* = 6
Gender (n)	Female	10	8	2
	Male	10	6	4
Age (years)	Mean	64.0	61.6	69.8
	Min-Max	46–80	46–80	65–75
Race (n)	White	13	13	N/A^a^
	Multiracial	1	1	N/A^a^
Ethnicity (n)	Not Hispanic or Latino	12	12	N/A^a^
	Hispanic/Latino	2	2	N/A^a^
Highest level of education (n)	Some high school	0	0	0
	Completed high school	8	4	4
	Some college	8	8	0
	Bachelor’s degree	3	1	2
	Not listed	1	1	0
Work status (n)	Full-time	7	7	0
	Part-time	1	1	0
	Disabled	1	1	0
	Retired	11	5	6
Time since T2D diagnosis (years)	Mean	17.4	16.7	19.3
	Min-Max	6.5–31	6.5–26	13–31
Time since first basal insulin treatment (years)	Mean	5.3	4.9	6.2
	Min-Max	0.5–18	0.5–18	3–13.5
Time since IDeg start (years)	Mean	1.1	0.6	2.4
	Min-Max	< 0.5–3	< 0.5–1	1.5–3

^a^ Race and ethnicity not collected in Switzerland for cultural reasons

IDeg, insulin degludec

Saturation analysis on the pooled data (*N* = 20) was reached with the 12th patient and plateaued until the 19th patient. At that point, 95% of the cumulative concepts were elicited. Two new concepts (pen noise and confusion) were elicited from the 19th patient who had socio-demographic and clinical characteristics that were diverse from most of the study population. She was one of two Latino/Hispanic patients in the study, was working full-time, and was younger than all other patients interviewed. We can conclude that saturation was met with a population with socio-demographic and clinical characteristics similar to the group’s average.

After discussions with T2D experts, four core benefits of IDeg were selected from the concepts found during the thematic analysis that were central to patients’ reasons for “feeling better”: 1) a reduced sense of diabetes as burdensome and requiring excessive attention, 2) enhanced feelings of adaptability and freedom, 3) a heightened sense of security, especially regarding concerns about hypoglycemia and 4) a greater sense of physical well-being (greater energy/less fatigue). These core benefits are elaborated in the following paragraphs and supported by sample quotes identified by country, site and patient number (eg, country-site number, patient #).

### Reduced sense of diabetes as burdensome and requiring excessive attention

The participants in the study identified the impact that IDeg had on both psychological, professional and social quality of life and reported the feeling of living life “without” T2D.

*“For me it's like I can do everything, I don't feel like I have* Type-2 *diabetes.”* (Swiss #08).

*“I have no problems with it at all and it allows me to live a normal life. I really don't feel like I'm terribly sick.”* (Swiss #02).

Patients cited benefits such as a more normal life experience with greater enjoyment and less stress. Some patients described being able to live without feeling like they are sick.

*“It really has helped me–like, say-at work-to concentrate better. I’m not battling the up and down. I can concentrate on everything, which is good.”* (US-01 #03).

*“It helps me enjoy the event-it helps me enjoy being around my friends and family. The stress factor is gone.”* (US-02 #01).

### Enhanced feelings of adaptability and freedom

Patients reported that when using IDeg, there were benefits to being able to adapt the dosing regimen to their daily schedule.

*“Also this pressure in case you forget it, I do everything in the morning now and then I can just live the rest of the day. I don't have to think about it anymore and for me this is a great relief.”* (Swiss #07).

*“I’m kind of I guess not real structured sometimes so having to do something at certain time of day and everything else just doesn’t always work for me. There’ll be times I’ll forget to take it like at 9 o’clock and all of a sudden it’s midnight or something like that, so that’s been really good. So I can kind of take it when I want.”* (US-02 #01).

Additionally, they felt there was greater freedom with regard to meals and IDeg use and expressed appreciation for not having strict time constraints with regard to administration.

*“[If I] wanted to eat some pizza-the next morning my sugar would be off the chart, and the way I’m going right now […] saying I could eat a whole pizza, and then […] the next morning, when I took the blood, you know-it’d [the blood sugar level] be right where it’s supposed [to be], so I really like the stuff.”* (US-01 #01).

### Heightened sense of security, especially regarding concerns about hypoglycemia

Blood sugar stability was an important concept noted that contributed to “feeling better.”

*“I was really scared because I once had low blood sugar levels on the highway, managed to stop on the service lane and passed out […] For me the aim was to never have low blood sugar again and in fact it never happened again with IDeg.”* (Swiss #02).

Participants indicated that IDeg helped them feel less anxious about their T2D, citing less concerns about the possibility of experiencing hypoglycemia and related consequences.

*“I’m not having to worry about the lows as much, because with the other medications-there were times where my levels would drop real fast, real low, and I would be driving …I never knew when those lows were going to hit, and especially driving, those could be very scary moments.”* (US-02 #01).

Participants expressed a greater sense of security when taking IDeg compared to previous basal insulins. Medication management–confidence patients feel in their medication’s ability to do its job and to deal with their treatment–was the second most frequently discussed concept.

*“There was no life quality at all, before every meal and first, you weren't allowed to eat anything in between […] by the time you had time to do it, it was too late and then I had to be scared to get low blood sugar levels, and I had to inject a certain amount of units according to a table, and if it wasn't right it didn't go well. And as a chaotic person I wasn't able to get a regular rhythm.”* (Swiss #03).

### Greater sense of physical well-being (greater energy/less fatigue)

Many patients reported a greater sense of physical well-being while using IDeg including greater energy and less fatigue.

*“A lot better. More energy […] I feel more like getting up and doing things […] I felt like looking at the country, and getting out, and trying to exercise.”* (US-01 #08).

In some patients, this resulted in being more active physically and also feeling more motivated to be active.

*“I had much more problems with this than I had before taking it. There is much more room for one-offs from a sporty point of view, in terms of performance I can do much more than before.”* (Swiss #08).

*“I’m restructuring my body, it’s helping me be more active actually.”* (US-01 #03).

### Findings by country

Although the recruitment numbers from Switzerland and the US do not allow for a robust comparison of findings by country, some interesting observations emerged. Blood sugar stability and confidence in the medication were the most important concepts, regardless of country. However, in general, Swiss patients had a more holistic view of the effect of IDeg on their lives; they were more likely to discuss their overall HRQoL than specific aspects of the medication. For example, Swiss patients often used broad statements such as “It’s like I can do everything” and expressed feelings of not having problems with T2D and living a normal life. Conversely, patients from the US were more likely to discuss specific benefits such as having more energy, being more active and productive, and worrying less about hypoglycemia. Further, US patients were much more likely to discuss their medical results; they could even cite their HbA1c and blood sugar levels from memory. For Swiss patients, medical results were not considered “markers” of T2D success as much as for US patients.

## Discussion

A continuing and important focus of diabetes management is improvement of HRQoL and how to achieve this goal [14]. Previous studies have observed improvements in HRQoL with IDeg use compared with insulin glargine [2–4]. However, there are little published data describing the nature of these effects. Prior findings reporting improvements in HRQoL while being treated with IDeg have relied primarily on data gathered using the 36-item short-form health questionnaire (SF-36) [2, 3] a tool frequently used in clinical trials, but which does not provide qualitative patient experience information to understand the nature of noted improvements. We used qualitative research methods to better understand the patient experience associated with IDeg in terms of HRQoL (well-being, functioning, social), activities of daily living, sleep/vitality and other issues that were revealed during the interview process. Additionally, we explored which attributes of IDeg contributed to the patient experience. Summarizing data from the focus groups, four core benefits of IDeg were selected from the themes and concepts. Patients reported an improvement in HRQoL resulting from a reduced sense of diabetes as burdensome and requiring excessive attention. They reported enhanced freedom due to the adaptability of dosing with regard to meals and their daily routine. Patients reported reduced anxiety and a sense of living life “without” T2D, reflected by expressions of not thinking about T2D as much during the day, less stress about T2D, and simply feeling more “normal.” Additionally, increased energy level and reduced fatigue reported by patients contributed to the sense of improved HRQoL. It is likely that less anxiety contributes to better sleep patterns and exercising more, although this remains to be confirmed.

Fear of hypoglycemia is a well-documented source of anxiety and emotional distress among patients with diabetes [15–17]. IDeg has been shown to have a lower risk of hypoglycemia compared with insulin glargine [18, 19], thus the improved HRQoL while using IDeg is likely due, at least in large part, to this phenomenon. A number of subjects in the current study specifically cited lessened fear about hypoglycemia while using IDeg. Hypoglycemic episodes of any severity might contribute to patient anxiety and lower HRQoL, even non-severe episodes which may be under-reported in clinical trials. However, the emotional impact related to non-severe hypoglycemic episodes, or a decrease in such, are likely to be reflected by HRQoL assessments [4].

Prior research has identified that feelings of powerlessness and not being able to do a good job of controlling diabetes are major contributors to poor HRQoL [17]. We identified that anytime daily dosing and adaptability with regard to food scheduling contributed to the improvement in the psychological HRQoL that patients described as a reduction in anxiety and the sense of “living life without T2D.”

Focus groups, which have been used in qualitative research since the 1940s, are often considered more cost and time efficient than interviews, given that they enable researchers to explore the opinions of many participants at once. That said, discussing research topics in a group setting has many methodological advantages as well. First, focus groups can act as a type of micro social world wherein the researcher can observe how interactions unfold. Valuable insights can be gained about the attitudes of participants and the negotiation strategies they use to reach a common decision or assert a particular belief [20]. Second, if a focus group’s participants are chosen carefully, the group can create a safe space in which participants can give voice to opinions or experiences that might be embarrassing or sensitive in the context of a one-to-one interview. For example, participants who have suffered from sexual abuse may be more likely to discuss it openly when they are sharing experiences with others who have the same history [21]. Third, focus groups help to reframe the role of the researcher. Often seen as an authoritative, dominant figure, in a focus group the researcher takes a back seat and acts more as a facilitator than in an interview, letting the participants create their own dialogue and tell their own stories [22].

Focus groups, like all methodologies, come with their own set of disadvantages as well. The most important of these is the silencing of non-conformist viewpoints. If participants are not chosen carefully and/or the researcher is not well trained, this outcome become a risk [21]. Second, focus group data can be difficult to transcribe and to analyze, as not all participants may express their thoughts in equal detail and many participants often speak at once. Researchers must therefore pay attention to encouraging all participants to speak and to preventing uneven group dynamics, such as the dominance of particular participants or the stigmatization of participants’ ideas which are not the norm [20].

The main limitation of the study was that it was a relatively homogenous sample. The sample was ethnically homogeneous (mostly white) and was not representative of the US (only Montana and California) or Swiss (only the Zurich suburbs) T2D populations. The sample was also biased in terms of employment status (mostly unemployed or retired) and age (> 60 years old). This is supported by fact that the last new concepts were introduced by a patient whose socio-demographic characteristics differed from the mean of the sample. To confirm saturation in a broader population, additional interviews would need to be conducted with characteristics similar to the 19th patient. Another potential limitation was that both phone interviews and focus groups were utilized which could have introduced a response bias. In addition, the study was not designed to assess issues with IDeg compared to other basal insulin types. It is important to note that the participants in this study were recruited specifically because of a reported positive experience on IDeg and were intentionally biased in that regard. Thus, while this sample served the purposes of evaluating reasons for a positive experience, further study is needed to determine whether the reported benefits are widespread among IDeg users and if these features are unique to IDeg relative to other basal insulins.

Despite the limitations of this study, the findings may be useful to help in the selection of appropriate psychometric instruments for future studies seeking to identify the potentially unique HRQoL benefits resulting from the use of novel diabetes medications such as IDeg.

## Conclusions

This group of patients with T2D, who reported “feeling better” after switching to IDeg from another basal insulin, communicated four major HRQoL benefits. First, patients expressed feeling less burdened by day-to-day diabetes demands. Second, while using IDeg, patients said they experienced increased adaptability and freedom in their day-to-day lives. Third, patients indicated feeling a heightened sense of security and less anxiety about hypoglycemia while using IDeg. Finally, patients reported broader (ie, not specific to diabetes) HRQoL gains such as higher energy and less fatigue. Although limited by the population of white, age > 60-year-old healthy subjects (without physical or mental comorbidity), these findings may be useful for future studies seeking to identify the potentially unique HRQoL benefits resulting from the use of novel diabetes medications such as IDeg. The conclusions may have limited transferability due to the characteristics of the sample population and further research is needed.

## Abbreviations

HRQoL: Health-related quality of life
IDeg: Insulin degludec
T2D: Type 2 diabetes
US: United States
